# Sonocatalysis: A Potential Sustainable Pathway for the Valorization of Lignocellulosic Biomass and Derivatives

**DOI:** 10.1007/s41061-017-0122-y

**Published:** 2017-03-23

**Authors:** Ewelina Kuna, Ronan Behling, Sabine Valange, Gregory Chatel, Juan Carlos Colmenares

**Affiliations:** 1 Institute of Physical Chemistry, Polish Academy of Sciences, Kasprzaka 44/52, 01-224 Warsaw, Poland; 2 Institut de Chimie des Milieux et Matériaux de Poitiers (IC2MP), Université de Poitiers, CNRS, ENSIP, B1, 1 rue Marcel Doré, 86073 Poitiers Cedex 9, France; 3 Univ. Savoie Mont Blanc, LCME, F-73000 Chambéry, France

**Keywords:** Sonochemistry, Sonocatalysis, Biomass upgrading, Lignocellulosic waste valorization, Lignocellulose depolymerization

## Abstract

**Abstract:**

Lignocellulosic biomass represents a natural renewable chemical feedstock that can be used to produce high value-added chemicals and platform molecules. Nowadays, there are extensive studies on a variety of aspects concerning the valorization of lignocellulosic biomass into desirable products. Among the current technologies for biomass conversion some require extreme conditions along with high temperatures and pressures. Therefore, major technological innovations based on more economical and environmental methodologies are currently developed both in academic laboratories and in industry. In this context, ultrasound-assisted catalysis constitutes an alternative method offering new strategies to upgrade biomass. The possibility of combining catalysis with sonication indeed provides avenues that are worth exploring for the valorization of lignocellulosic compounds into value-added chemical feedstocks. In this mini-review, the available sonochemical systems are first presented, with a focus on the most important ultrasonic parameters, which is intended to provide a mechanistic background. Next, this contribution aims to provide insight into the most recent developments along with prominent examples in the field of sonocatalysis applied to the chemical transformation of lignocellulosic biomass and its derivatives.

**Graphical Abstract:**

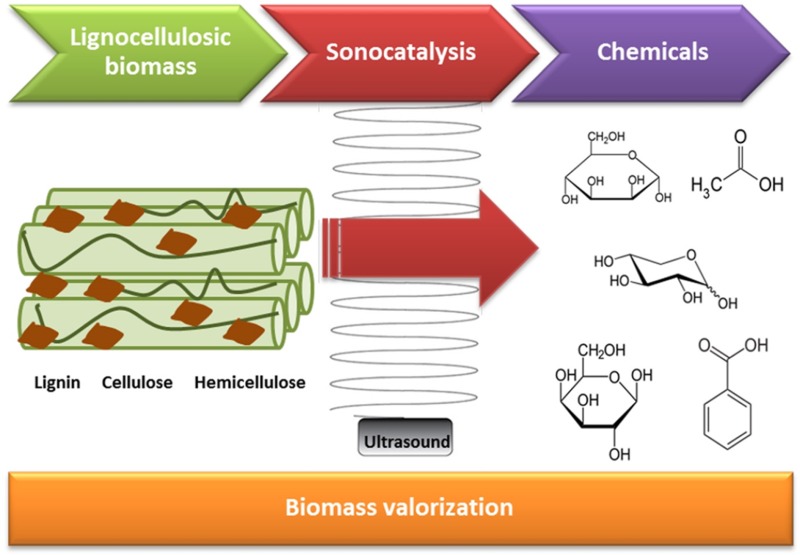

## Introduction

**Fig. 1:**
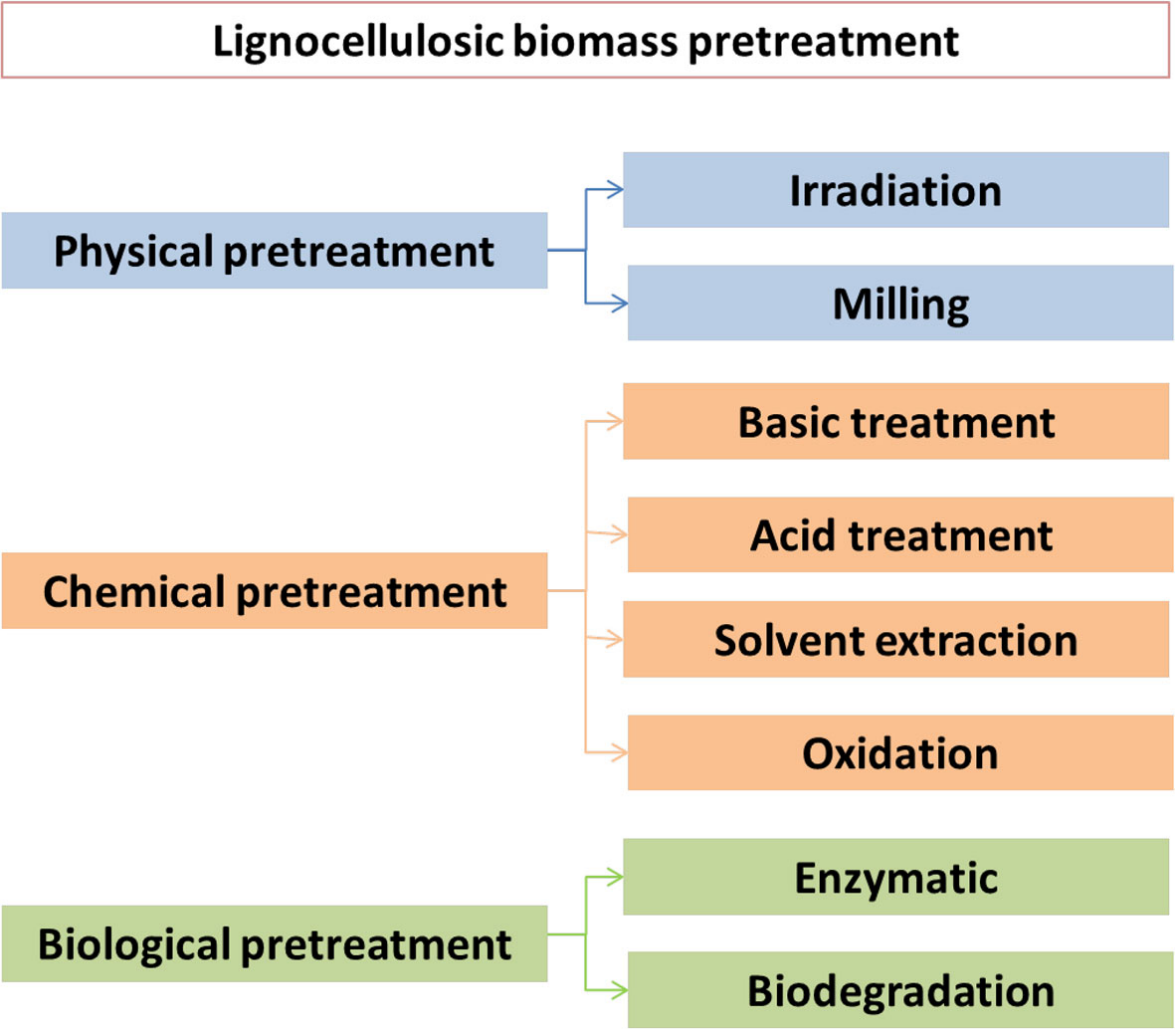
Methods for pretreatment of lignocellulosic biomass. (Adapted and modified from Ref. [9])

The worldwide accessibility of biomass undoubtedly plays a fundamental role in sustainable energy production. In favorable circumstances, around 25% of global energy requirements can be supplied by biomass [1]. The enormous potential of plant materials resides in the presence of lignocellulosic residues coming from forest residues, energy crops, municipal and industrial wastes [2]. This type of renewable resource is now increasingly used for the production of value-added chemicals and transport fuels [3]. Nevertheless, plant dry matter possesses a sturdy structure, which complicates the effective conversion process of lignocellulosic biomass into platform chemicals. Lignocellulose is a recalcitrant biopolymer composed of the semi-crystalline polysaccharide cellulose, the polysaccharide hemicellulose, and the three-dimensional amorphous phenylpropanoid lignin polymer [4, 5]. The conversion of lignocellulosic feedstock leads to the production of a variety of added value platform chemicals, including phenolic compounds (*p*-coumaryl alcohol, coniferyl alcohol, and sinapyl alcohol), aliphatic acids (formic acid, acetic acid, levulinic acid), and furan aldehydes (hydroxymethyl furfural) [6]. It is worth mentioning that the accessibility to desired products is dependent on biomass pretreatment methods, which have a significant influence on the production of their derived feedstocks for further valorization strategies. Notwithstanding, the selection of the most favorable pretreatment process depends predominantly on the target molecule and many other factors such as economical and environmental aspects [7]. The depolymerization process of lignocellulosic biomass includes physical, chemical, and biological treatments, as well as various combinations thereof (Fig. 1) [7–9]. The physical pretreatment of biomass as a first step for further upgrading is achieved by mechanical comminution of lignocellulosic materials through a combination of chipping, grinding, or milling. In the particular case of cellulosic biomass, such physical process leads to a size reduction due to a decrease of both the degree of polymerization and the crystallinity, resulting in the increase of the mass transfer and the improvement of the hydrolyzation reaction. However, the energy consumption required for physical treatment is higher than the theoretical energy content available in lignocellulose, which makes it prohibitively expensive for large-scale uses [10]. An alternative for deconstructing lignocellulosic biomass is chemical pretreatment. This method is based on catalytic processes such as acid/alkaline hydrolysis, oxidative delignification, cracking, reduction reaction, among others [11]. Acid pretreatment allows converting hemicellulose into monomeric sugars (e.g. glucose, xylose) and soluble oligomers, whereas alkaline hydrolysis renders lignin recoverable. Delignification of lignocellulosic biomass can also be performed by treating in the presence of oxidizing agents (e.g. hydrogen peroxide, ozone, and oxygen) [12]. In most of these cases, catalysis increases the efficiency of the process and is responsible for the major effects achieved by pretreatment [13]. The application of catalysts provides a more effective approach in biological processing, where the yield of hydrolysis is relatively low along with long pretreatment times. Enzymatic hydrolysis can also be improved by combining ultrasonic pretreatment with the organosolv process. Ultrasound has the potential to enhance the separation and hydrolysis of lignocellulosic materials [12]. By contrast, the combined use of organic solvents (including ionic liquids) or their mixtures with water was shown to enhance dissolution of biomass and increase depolymerization rates [14].

**Fig. 2:**
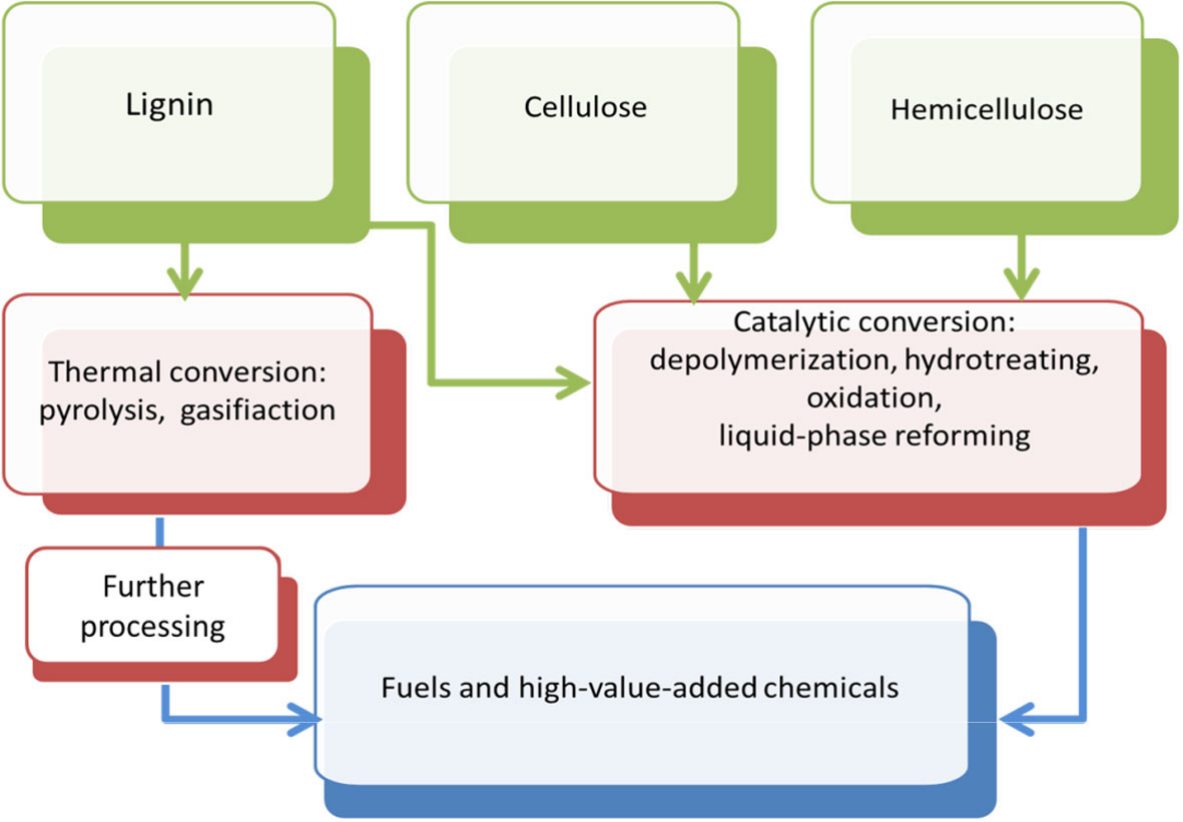
Biorefinery strategies for lignocellulosic biomass valorization to fuels and chemicals. (Adapted and modified from Refs. [8, 17])

The immensity of available pretreatment methods creates opportunity to choose those which enable downstream processing of lignocellulosic biomass. Recovered carbohydrate polymers such as cellulose and hemicellulose can further be transformed into fermentable sugars and then into fuels or feedstock chemicals. Valuable products can also be obtained from lignin through development and integration of current and new technologies such as sonocatalysis, heterogeneous photocatalysis, or microwave-assisted conversion [15, 16]. According to a classification proposed in previous papers [8, 17] a simplified summary of conversion strategies is given in Fig. 2. Thermal technologies can be used to produce solid feedstocks (e.g. biochar), liquids (e.g. oils and viscous tars), and gaseous products. However, methods such as pyrolysis and gasification require large energy inputs due to the high processing temperatures. Chemical conversion techniques (e.g. catalyzed depolymerization, hydrotreating, oxidation, liquid-phase reforming) constitute a more energy efficient and environmentally sustainable way to valorize biomass [17].

In the present review, we focus on the recent literature advances on sonocatalytic valorization of lignocellulosic biomass and their platform molecules. It is evident that catalysis is regarded as a key route enabling technology for pretreatment and conversion of lignocellulosic biomass [8]. Over the past few years, research on the development and optimization of highly active and selective catalytic systems has been an ongoing activity to overcome drawbacks associated with harsh chemical conditions, low yield production, and high processing cost [18]. Additionally, sonochemical-assisted reactions offer opportunities to develop environmentally friendly and cost-effective processes for biomass upgrading [4, 19].

## Generalities on Sonochemistry

Sonochemical effects arise from cavitation, which is defined as the phenomenon of formation, growth, and implosive collapse of bubbles under the influence of an ultrasonic field in liquids [20, 21]. Cavitation can be categorized into various forms (acoustic, hydrodynamic, optic, and particle cavitation) depending on the method of generation and associated ultrasonic/experimental parameters (frequency, acoustic power, shape of the reactor, solvents, temperature, pressure, etc.). Acoustic and hydrodynamic cavitation may generate physical and chemical changes in solution in contrast to optic and particle cavitations. Numerous “hot spots” can be created by acoustic and hydrodynamic cavitation due to the accumulation of a huge amount of energy which in turn results in immense pressures and temperatures [22, 23]. The pressure fluctuation induced by changing the geometry of the flow system produces hydrodynamic cavitation, while the pressure fluctuation in the passageway of sound waves induced acoustic cavitation [24].

**Fig. 3:**
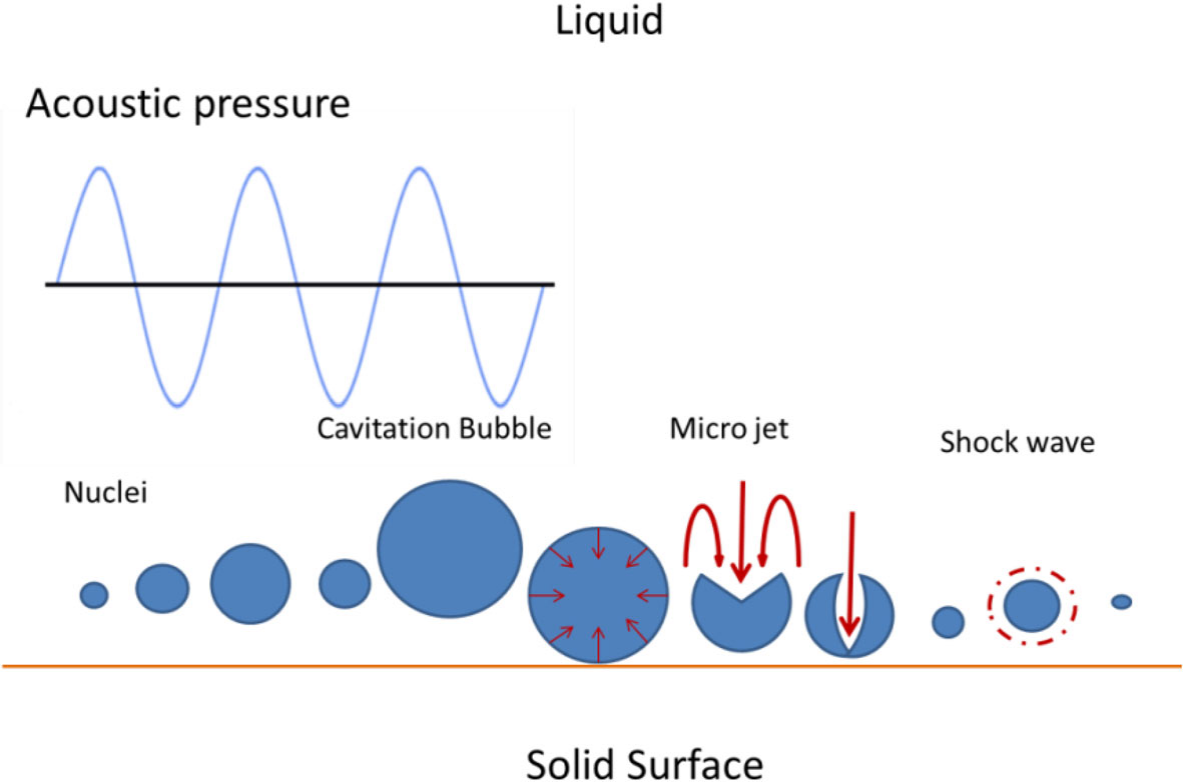
Acoustic cavitation mechanism

Acoustic cavitation takes place within collapsing bubbles (gas-phase chemistry), on the outer side of the bubbles (solution-phase chemistry) and at the liquid–solid interface (physical modification) [21]. The chemical and physical effects of ultrasound eventuate from the cavitation phenomenon and not from direct interaction between chemical species and ultrasonic waves [21, 25]. The chemical effect of ultrasound is the consequence of the implosive collapse of microbubbles, producing free-radicals, whereas the physical effects are the result of shock waves and microjet generated during symmetric and asymmetric cavitation, respectively (Fig. 3) [26].

The rate of motion of ultrasound is significantly greater than the molecular scale [23]. Generally, when the sound velocities in a liquid are around 1000–1500 m s^−1^ the power ultrasound (in opposition to diagnostic ultrasound, particularly used in medical imaging) can oscillate from approximately 10 to 10^−4^ cm over the frequency range of 20–2000 kHz [21, 22]. The compression and expansion waves put the liquid under dynamic tensile stress. As a result, the local pressure decreases adequately below the saturated vapor pressure and initiate cavitation [21, 27]. Microbubbles present in a liquid absorb energy from ultrasound waves and undergo a rapid overgrowth leading to violent collapse. The final stage of implosion is almost adiabatic and provides extreme conditions [21, 28].

### Homogeneous and Heterogeneous Sonochemical Systems

**Fig. 4:**
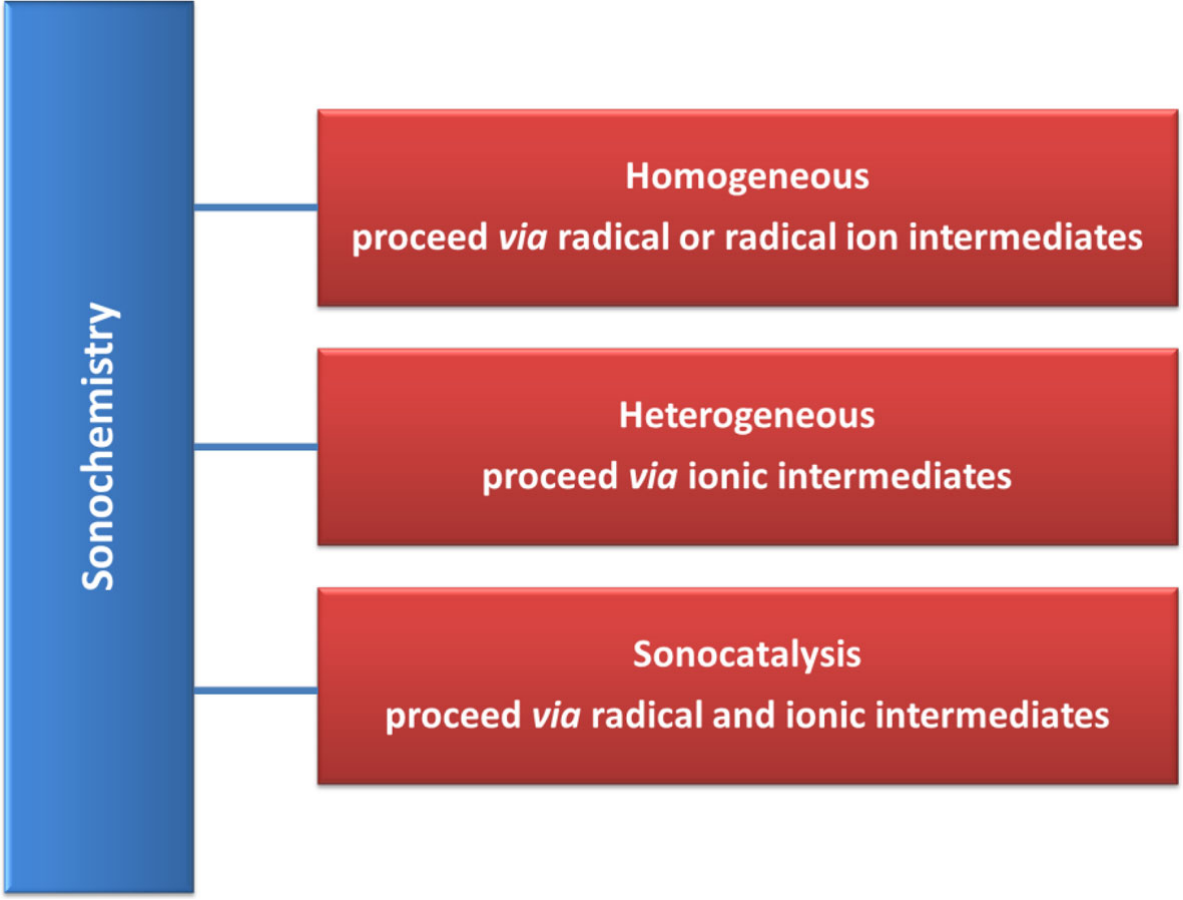
Classification of sonochemistry reactions

Sonochemical reactions can be classified into three categories, namely homogeneous sonochemistry of liquids, heterogeneous sonochemistry of liquid–liquid or liquid–solid systems, and sonocatalysis (which involves the aforementioned systems) (Fig. 4) [29, 30]. Homogeneous systems include radical reactions, which are accelerated by sonication and that follow via radical or radical-ion intermediates [32]. In this case, the chemical bonds are broken under the high temperature and pressure generated during cavitation [24]. The short-lived chemical species are turned back to the bulk liquid and react with other species [32]. Sometimes homogeneous sonochemistry followed by secondary reactions taking place in the liquid, especially in the case of compounds of low volatility, which can interact with radical species produced from solvent sonolysis [33]. In homogeneous systems, where the surroundings are uniform, the cavity remains spherical. Cavity collapse in heterogeneous system may proceed via two fundamentally different mechanisms such as microjet impact and shock wave damage. Deformation in the cavity is caused by asymmetric motions of the molecules in liquid during cavity collapse. The expanded bubble’s potential energy is converted into kinetic energy of a high-speed liquid jet that passes through the bubble’s interior and pierces the opposite bubble wall. The available energy is predominantly handed over to the accelerating jet rather than the bubble wall itself [34, 35]. High energy concentration can cause an intense damage to the boundary surface. The stress fracture on the surface can be invoked by shockwaves generated through cavity collapse in the liquid. The impingement of microjets and shockwaves form the localized erosion, which is in charge of ultrasonic cleaning and another sonochemical effects such as particle size reduction or improved mass transfer on heterogeneous reactions [24]. In heterogeneous systems, the use of ultrasound accelerates chemical reactions, drawing on mechanical effects of cavitation. The dynamics of the cavity collapse changes dramatically when cavitation takes place in a liquid nearby a solid surface [32]. The imposition of the heterogeneous and homogeneous sonochemistry includes a radical and an ionic reaction mechanism. Indeed, depending of the ultrasonic frequency (see Sect. 2.2), the sonication enhances radical formation and mechanical effects (e.g. mass transfer) [30].

### More Important Parameters in Sonochemistry

The selection of ultrasonic parameters (such as frequency, acoustic power, temperature, pressure, solvent, design of reactors, etc.) is a crucial issue in order to optimize the system and thus influence efficiency of a chemical process. There are different ways to optimize these parameters depending on the target experimental outcome; they can be chosen by taking into account the data concerning studies on bubble cavitation characteristics [36]. The problem connected with cavitation distribution and quantification might be addressed by using different experimental or theoretical mapping techniques [37]. The basic aspects of each method, their applicability, pros and cons have been highlighted by Sutkar and Gogate [37, 38]. Their analysis allows one to determine the behaviour of cavitational activity regarding reactor geometry over a range of operating parameters [24]. In order to estimate mutual relation, bubble dynamics analysis has been also employed by other researchers [37, 39]. The study includes quantifying the correlation between the pressure and temperature linked with cavity implosion as a function of intensity, frequency, and initial radius of the nuclei [24].

The cavity dynamics is defined by two elements: the maximum magnitude accomplished by the cavity before implosion (that determines the pressure and temperature generating during collapse) and the lifetime of the cavity (that defines the distance travelled by the cavity from the place where it is formed) [37, 40]. Both elements are of paramount importance for the design of sonochemical reactors and should be optimized by appropriately adjusting the various geometric and operating parameters [37]. The reactor design has significant effects on the cavitational activity, in terms of the reactor and horn tip diameters (including the ratio of both diameters) and the position of the horn tip immersed in the liquid [24, 41].

Another factor that has an influence on the cavitation is the amount of energy, which is supplied to the bulk solution. The power dissipation rate varies on the extent of temperature growth, which causes direct changes in the gas solubility and vapour pressure, generating active cavitation sites. Energy efficiency is expressed by the amount of energy dissipated into the liquid and is generally calculated by a calorimetric method (monitoring the temperature as a function of time allows to estimate the acoustic power) [24, 37].

The effect on the bubble cavitation is directly connected to the frequency of the ultrasound. We can distinguish frequencies at low range (20–80 kHz) and high range (>150 kHz) [42]. High frequency does not promote the occurrence of active cavitation, because of insufficient duration of the ultrasonic cycle, which is required for the growth, radial motion, and collapse of bubbles. The short-lived bubble can boost the concentration of free radicals and may have a higher probability to get out the cavitation site to the bulk mixture. Compared to lower frequency ultrasound, high ultrasonic frequencies produce less violent cavitation and lead to chemical effects. Low frequencies are responsible for physical effects, where rapid cavitation leading to enormous temperatures and pressures at the cavitation site [43]. It is noteworthy that physical properties of the liquid phase have also many effects on ultrasonic cavitation. Relative low volatility, viscosity, and high surface tension of liquid solvents are preferred for favoring efficient cavitation [37]. Active cavitation occurs also in heterogeneous sites in liquids such as impurities, gas microbubbles, non-volatile additives, etc. [36].

The physical and chemical effects produced by ultrasonic cavitation in a liquid phase provide extreme local conditions such as immense local heating (~5000 °C), pressures (~1000 atm), and heating/cooling rates (10^10^°C s^−1^) [34]. Microjet streams and shock waves created by cavitation promote better energy and mass transfer, which has an impact on accelerating chemical reaction, increasing conversion, improving the yield, and enhancing the selectivity in both homogeneous and heterogeneous systems [44]. For this reason, sonochemistry has found wide applications in chemical synthesis used for the preparation of nanostructured materials (e.g. hybrid lignocellulosic materials) [45] and modification of inorganic materials (e.g. clay minerals) [46]. Additionally, the benefits of the use of ultrasound in organic synthesis are also highlighted by interesting review articles [47–49]. Some key examples where synthetically useful sonochemical organic transformations carried out in homogenous and heterogeneous conditions include: hydrolysis [50], cycloaddition (e.g. Diels–Alder reaction) [51], coupling (e.g. Suzuki reaction, Mitsunobu reaction) [47], isomerization (e.g. glucose to fructose) [52], alkylation [53], esterification [54], and polymerization reactions [55].

## Ultrasound-Assisted Catalysis for Lignocellulosic Biomass Valorization

### Principles of Sonocatalysis

**Fig. 5:**
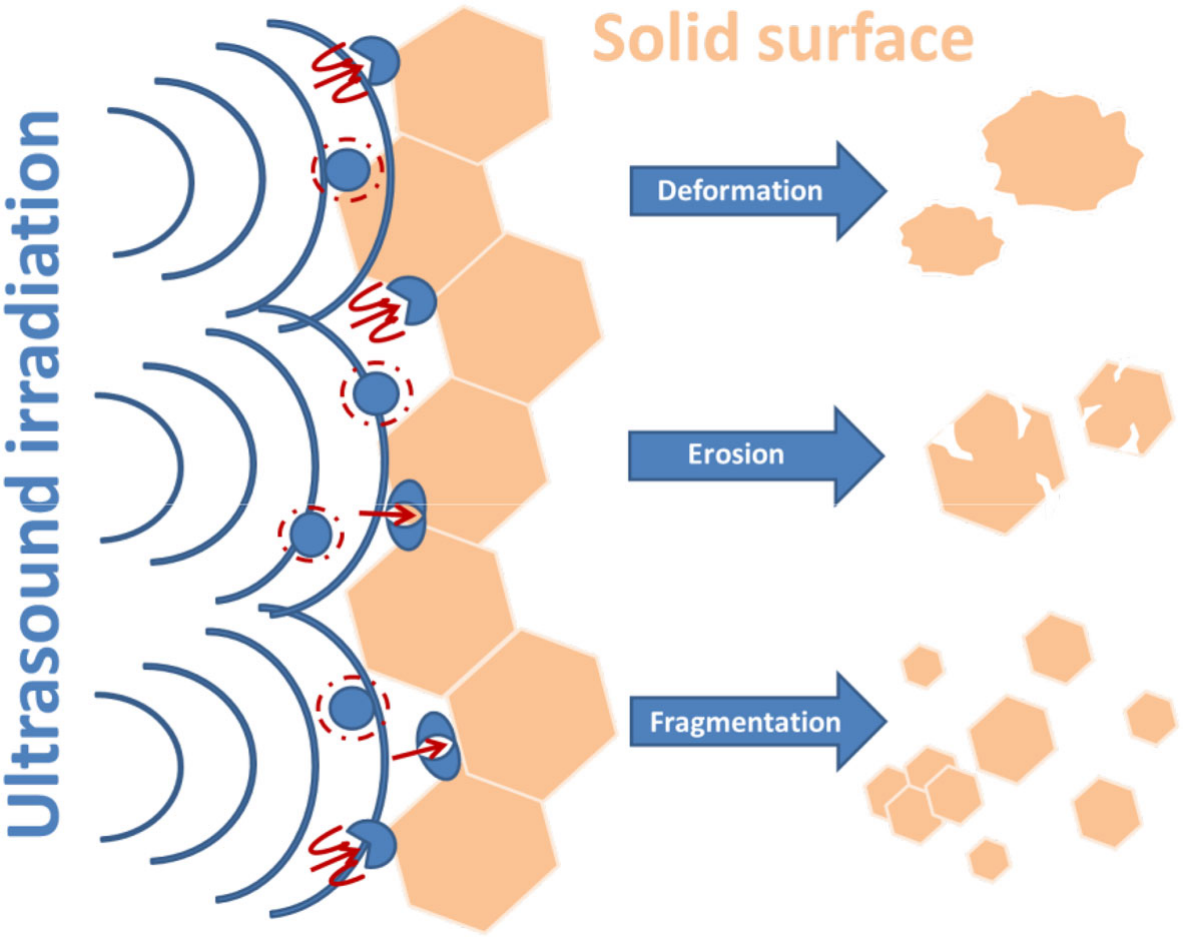
Effect of ultrasound irradiation on a heterogeneous catalyst surface

The combination of sonochemistry with catalysis can be used to accomplish a number of chemical reactions with convenient workup conditions (e.g. shorter reaction times) in contrast to more conventional methods [56]. Heterogeneous reactions follow via ionic intermediates provoked by mechanical effects, whereas radical reaction enhanced mainly by sonication. In the case when radical and ionic mechanisms lead to other products, ultrasound might promote the radical reaction, which can also provide new synthetic pathways [30]. The fundamental rule of sonocatalysis is diffusion and sorption of the main components on a solid surface followed by a series of heterogeneous chemical reactions on active sites [57]. In a heterogeneous reaction system, the improvement of chemical reaction is mainly caused by physical effects. The physical phenomena improve mass transfer from turbulent mixing and acoustic streaming, generate cavitation erosion at liquid–solid interfaces, and are responsible for deformation of solid surfaces (Fig. 5) [25].

The effect of ultrasonic irradiation on a heterogeneous catalyst may cause physical and chemical modifications (e.g. changes in crystallization, dispersion, and surface properties, as well as changes on catalytic reactivity during reaction (Fig. 5) [58]. The chemical rate increases due to enhancement of external transport phenomena and the increase in temperature at the catalyst surface. Acoustic cavitation can induce the breaking of the catalytic particle and gives more accessibility to the internal surface for the reagents. In the gas–liquid-solid system (e.g. hydrogenation reactions) sonication increases the interphase surface and favors the removal of outer oxide or other passivating layers from the catalyst surface [49].

### Homogenous and Heterogeneous Sonocatalysis

The application of ultrasound in homogeneous and heterogeneous reaction systems in the presence of catalysts is viewed as a convenient technique for lignocellulosic valorization. Catalysis assisted by ultrasound includes a variety of reactions such as hydrolysis, hydrogenation, oxidation [59]. Sonication improves hydrolysis of lignocellulosic materials into sugars and their subsequent fermentation into bio-ethanol. The main reason for enhanced conversion is the substantial improvement of mass transfer in reacting systems, as well as the activation of chemical and biological catalysts [37]. Yunus et al. reported that the acid hydrolysis of palm oil to xylose was increased from 22 to 52% under ultrasound pretreatment (20 kHz, 2 kW), in comparison to silent conditions (Table 1; Entry 1) [60]. An improvement of the reaction rate under ultrasound (25 kHz, 600 W) was noticed also by Choi and Kim during the acid-catalyzed hydrolysis of starch (Table 1; Entry 2) [61]. Ultrasonic energy can have a direct influence on hydrolysis and fermentation reactions of cellulosic materials, while the application of ultrasound with enzymes accelerates saccharification and the fermentation rate [36]. Cavitation effects enhance the transport of enzyme macromolecules to the surface of the substrate, whereas the substrate surface is opening up to the action of enzymes due to the mechanical effect of cavitation [4, 62]. Additionally, the sono-assisted enzymatic conversion of cellulose performed in solvents such as ionic liquids (ILs) yielded high performances by promoting high conversion, yield, and selectivity [36]. The combination of ultrasound with ILs has indeed recently attracted much interest for lignocellulosic biomass valorization [36]. For example, enzymatic hydrolysis of lignocellulose assisted by ultrasound (45 kHz, 100 W) in imidazolium-based ionic liquid media improves cellulosic conversion from 75 to 95% [63] and saccharification ratio to 92% from 55% [64] in 60 min (24 kHz, 35 W). The benefits of ultrasound-assisted enzymatic processes are the enhanced solvation and the increased reactivity of biomass reactants, coupled to reactions taking place at lower temperature within shorter time and with less requirement for acid or base catalysts [36].

**Table 1:** Homogeneous and heterogeneous sonocatalytic biomass valorization

Entry	Process	Catalyst	Substrate	Sonochemical parameters	Experimental details	Sonocatalytic behaviour	References
1	Hydrolysis	2% sulphuric acid (aqueous solution)	Oil palm empty fruit bunch	2 kW (20 kHz)	Reaction time 15, 45, 60 min Solvent H_2_SO_4_ concentration (2%) Temperature 25 °C	The highest yield of xylose (58%) was obtained under ultrasound irradiation (90% amplitude) during 45 min, whereas without ultrasonic pretreatment yield was equal to 22%	[60]
2	Hydrolysis	1–5% sulphuric acid (aqueous solution)	Starch Maltose Maltotriose	600 W (25 kHz)	Reaction time 100 min. Solvent dilute sulfuric acid (1–5 wt%) Temperature 90–100 °C	The reaction yield in the presence of ultrasound is higher than in the reaction without sonication (increased ~75 % at 90 °C)	[61]
3	Hydroprocessing	Fe_3_O_4_ (NiAlO) x Fe_3_O_4_ (NiMgAlO) x	Miscanthus (lignin, glucose, xylose, arabinose, galactose, mannose, extractives, and ash)	(35 kHz)	Reaction time: 6 h/24 h. Solvent: ethyl acetate/methanol/ionic liquid [BMIM]OAc Temperature room temperature/180 °C	Catalysts exhibited a slight activity whereas considerable growth in conversion (up to 90% under US irradiation) was noticed on nano-Ni (0) and NiO (111) nanosheets samples	[66]
4	Degradation	Rutile- TiO_2_ Anatase-TiO_2_ Montmorillonite Clay (MMT), ZnO and Fe_3_O_4_	2-hydroxyethyl cellulose	100 W (24 kHz)	Reaction time 100 min Solvent water Temperature 25 °C	The sonocatalytic activity (rate constants) of nanoparticles catalysts increases in the following order: Fe_3_O_4_, Rutile-TiO_2_, ZnO, Anatase-TiO_2_, and MMT	[68]
5	Sonophotocatalytic Degradation	TiO_2_	Chitosan	30–90 W (24 kHz)	Reaction time 60 min Solvent water Temperature: 25 °C	In 60 min (at loading catalyst: 0.1–0.6 g/L) rate constant is modified in the range of 0.354–1.134 (mol^1.7^ L^−1.7^ min^−1^ × 10^−9^) while for the sonocatalytic degradation from 1.737 to 2.654 (mol^1.7^ L^−1.7^ min × 10^−9^)	[69]
6	Hydrolysis	Hydrochloric acid	Corn starch	Not provided by the authors	Reaction time 90 min Solvent hydrochloric acid Temperature 100 °C	The optimum conditions (levulinic acid yield 23%) were achieved during first 90 min at 100 °C with acid concentration (4.5 mol/L) and the ratio of liquid: solid (15:1 mL/g)	[72]
7	Oxidation	FeCl_3_/HNO_3_ (various Lewis acids)	A mixture of benzyl alcohols	120 W (35 kHz)	Reaction time 10–25 min Solvent acetone Temperature room temperature.	Ultrasound enhances chemical reactions and allows to obtain the complete conversion of benzyl alcohol into aldehyde during first 10 min reaction. However, continuation of the reaction over 20 min does not improve the yield (94%) and leads to other oxidized byproducts (e.g. benzoic acid)	[73]
8	Oxidation	TEMPO/NaBr/NaClO	Cotton linter pulp	300 W (40 kHz)	Reaction time 24 h Solvent water Temperature 25 °C	Ultrasonic-assisted by TEMPO oxidation allows obtain high carboxylate content (1.66 mml/g) cellulose nanocrystals (the widths 5–10 nm; the lengths 100-400 nm)	[74]
9	Oxidation	Au/SiO_2_	d-glucose	(35 kHz)	Reaction time 60 min Solvent: 30% hydrogen peroxide Temperature room temperature	The application of ultrasound provides high conversion (~100%) and selectivity (~80% into gluconic acid) and eliminates consequent reaction by products (fructose, mannose, glycolaldehyde, sorbitol, and maltose)	[79]
10	Hydrogenation	Raney-Ni Cu/SiO_2_ Cu/ZnO/Al_2_O_3_	d-fructose	0–50 W	Reaction time: 120 min Solvent − Temperature 70–110 °C	In the case of Raney-Ni catalyst, reaction selectivity was slightly lower at 30 W (47%) than under silent conditions (51%) and reactions carried out with 10 W (50%) and 50 W (50%)	[80]

The nature of solvents fulfils a crucial role in the lignocellulose depolymerization process. In some cases, ionic liquids were used only selectively to dissolve lignin rather than hemicellulose or cellulose [65]. For example, the catalytic hydroprocessing of lignin under ultrasound conditions resulted in a higher efficiency when conducted in an ionic liquid (1-butyl-3-methylimidazolium acetate) in comparison to organic solvents and water. Considerable enhancement in conversion up to 90% was noticed with nano-Ni and NiO nanosheets catalysts [66]. The conversion rate and the mass distribution of products depends on the procedure used for the pretreatment (acidic or alkali) of lignin. This means that in most cases, acidic extraction leads to a larger extent of depolymerization reaction (Table 1; Entry 3) [66]. Additionally, work by Napoly and co-workers shows that it is possible to obtain vanillin-based monomers with yield equal to 0.51 wt% in the presence of a tungsten-based catalyst and an oxidizing agent such as H_2_O_2_ [67]. Na_2_WO_4_ acted as the most promising catalyst, which promoted an effective system, where ultrasound generated sufficient oxidation conditions (20 kHz, 11 W) and involved strengthened oxidative coupling of phenoxy radicals [67].

A relevant study about the degradation of cellulose was recently published by Taghizadeh (Table 1; Entry 4) [68]. The degradation behaviour of 2-hydroxyethyl cellulose was conducted in the presence of a variety of heterogeneous catalysts (such as titanium oxide, montmorillonite clay, zinc oxide, and iron oxide) under ultrasound irradiation (24 kHz). Sonolytic degradation (without catalyst) increases with increasing of ultrasonic power (in the range of 30–90 W); however, it is remarkably lower in comparison to the efficiency of the sonocatalytic degradation. The results obtained revealed that the combined use of catalysts and US irradiation improved the degree of cellulose depolymerization. Among all the catalysts tested, the most efficient was Fe_3_O_4_, which provided a better ability for radical generation through electron transfer between the metal ion and the water molecules during the sonication process. The possibility of combining ultrasound irradiation with heterogeneous photocatalysis was also studied by the same research group (Table 1; Entry 5) [69]. The complete degradation of chitosan (with cellulose-based structure) was achieved during 1 h in the presence of titanium oxide at 24 kHz. In this case, sonophotocatalysis enhanced the production of reactive radicals as well as increasing the active sites of the catalyst surface.

Behling et al. recently investigated the low frequency (20 kHz) ultrasound-assisted aqueous-phase oxidation of vanillyl alcohol to vanillin using a heterogeneous Co_3_O_4_ catalyst with hydrogen peroxide as the primary oxidizing agent under mild reaction conditions (low temperature and atmospheric pressure). The outcome of this work was that the ultrasound-assisted catalytic reaction is faster (4x), more selective (2.3x), and more efficient (2.7x) than the corresponding reaction carried out under silent conditions [70]. Additionally, a large decrease of the overall energy consumption was observed under ultrasound (36  vs. 288 kJ). Moreover, from an environmental point of view, green metrics indicators such as the E factor and the process mass intensity (PMI) calculated for both activation systems clearly showed the benefit of the ultrasound-mediated reaction [70]. The ultrasound-microwave assisted process is also an interesting approach for lignocellulosic biomass valorization. The simultaneous microwave (100 W) and ultrasound irradiations was shown to improve the hydrolysis reaction rates of glucose [71] and corn starch [72] (Table 1; Entry 6) in 60 min. In both cases, the reaction yield to levulinic acid was high (49 and 23% from glucose and corn starch, respectively) in comparison with those reported in the open literature.

Kardos and Luche [42] have investigated interesting approaches to obtain high value-added chemicals through the conversion of biomass feedstocks such as polymeric carbohydrates to lower weight molecules. In the case of polysaccharides, the partial or total depolymerization has to be taken into account. Hydrolytic procedures, already mentioned before, have been widely examined to accomplish this aim. Nevertheless, particular attention should be paid to oxidation reactions. For example, glucose being selectively oxidized into glucuronic acid in the presence of iron sulfate under ultrasound irradiation (100 kHz), whereas hexoses are oxidized to the corresponding uronic acids. It is interesting to note that this type of reaction cannot be performed without oxygen or acoustic activation [42].

The sonocatalytic oxidation of primary benzyl alcohols into the corresponding aldehydes was reported by Naik et al. [73]. They noted that the mixture of HNO_3_/FeCl_3_ provides high yields (80–94%) of aldehydes within 10–25 min (Table 1; Entry 7). Reactions carried out under silent conditions showed fourfold lower rates and yields than those performed under sonication. The application of ultrasound (35 kHz, 120 W) gave excellent yields with short reaction times and allowed to avoid over-oxidized products.

The work of Qin et al. [74] showed that the TEMPO/NaBr/NaOCl oxidation system assisted by ultrasound (40 kHz, 300 W) can be used to prepare cellulose nanocrystals with high carboxylate content from cotton linter pulp (Table 1; Entry 8). This is consistent with the study carried out by Brochette–Lemoine et al. [75]. The results of these investigations indicated that the rate of the oxidation of methyl α-D-glucopyranoside or sucrose was increased in the presence of ultrasound. Moreover, the reaction can then occur without the commonly used sodium bromide owing to the ability of ultrasound to generate the active oxidizing species during the catalytic cycle. Additionally, sonication accelerated the oxidation reaction, especially when the frequency of ultrasound was increased from 20 up to 500 kHz [76]. In order to scale up the oxidation of cellulose, Paquin et al. [77] proposed the use of a continuous flow-through system instead of the classical standard batch mode. The flow-through reactor increased the reaction rate (~36%) in comparison to the batch reactor while decreasing the overall energy consumption (~87%) [4].

Another reaction worth interest for the industry is the production of gluconic acid from d-glucose. Rinsant et al. [78] have described a way to selectively oxidize glucose via a sono-Fenton process with hydrogen peroxide in the presence of iron (II) sulfate as catalyst. In contrast to preconceived ideas, they proved that sonochemistry does not constitute an “intensive energy consuming” technology. Furthermore, the energy consumption could be minimized when ultrasound-based processes are optimized. Energy consumption (under ultrasound) was lower than that attained under conventional reactions. Remarkably high conversion (~100%) and selectivity (~95%) values were obtained only after 15 min (20 kHz, 0.25 W mL^−1^). This example on d-glucose oxidation relies on the efficient combination of an eco-friendly oxidant (hydrogen peroxide) and ultrasound, which constitutes a promising strategy for the valorization of biomass. In the same strategy of sugar oxidation by sonocatalysis, Bujak et al. [79] observed that silica-supported gold catalysts are extremely active and selective for d-glucose oxidation to gluconic acid at ambient temperature and under ultrasound conditions (35 kHz) (Table 1; Entry 9). The application of ultrasound is of crucial importance to provide not only high conversion of glucose into gluconic acid with 100% yield, but also high reproducibility.

The effect of sonication (26, 78 and 130 W cm^−2^) on d-fructose hydrogenation in the presence of heterogeneous catalysts was examined by Toukoniitty et al. [80]. The reaction rate and selectivity were investigated at various conditions of temperature (70–110 °C), pressure (10, 30, and 50 bar), and ultrasonic power (0–50 W) (Table 1; Entry 10). The application of sonication during the hydrogenation reaction considerably accelerated the reaction rate in the presence of the Cu/SiO_2_ catalyst. High temperature and pressure had a moderate effect on the catalyst activity whereas the variation of nominal ultrasonic power input effectively improved the reaction rates.

## Challenges and Future Perspectives

In most cases, combining catalysis with sonication has interesting effects on reactions course. In the present mini-review, we have shown that the use of sonocatalysis allows avoiding harsh chemical conditions, along with reducing reaction times and improving heat and mass transfer, thereby increasing chemical rate constants, yields, and selectivities. Hence, the ultrasound-assisted catalysis can be successfully applied for the pretreatment and chemical conversion of lignocellulosic biomass and its derivatives in a variety of processes such as hydrolysis, oxidation, and hydrogenation reactions. The recent studies on the use of ultrasound to assist catalytic reactions have clearly shown great advantages and technological potential of this concept for the chemical industry, especially when thinking about processes under flow. More significant scientific breakthroughs for biomass valorization are expected to occur in this innovative field in the near future. Last, but not least, and continuing in the same lines, the important role of ultrasound on photocatalysis (ultrasound and photocatalysis together) for the valorization of lignocellulosic biomass and its derivatives might be also a promising research avenue worth broad interest in the huge spectrum of possibilities offered by lignocellulose-based processes, for instance, sonophotocatalytic proof concepts for: lignocellulosic biomass depolymerization [81], biohydrogen [82], and biomethane production [83].
